# A Brief History of Microglial Ultrastructure: Distinctive Features, Phenotypes, and Functions Discovered Over the Past 60 Years by Electron Microscopy

**DOI:** 10.3389/fimmu.2018.00803

**Published:** 2018-04-25

**Authors:** Julie C. Savage, Katherine Picard, Fernando González-Ibáñez, Marie-Ève Tremblay

**Affiliations:** ^1^Axe neurosciences, Centre de Recherche du CHU de Québec – Université Laval, Québec City, QC, Canada; ^2^Département de médecine moléculaire, Université Laval, Québec City, QC, Canada

**Keywords:** microglia, ultrastructure, electron microscopy, correlative light and electron microscopy, 3D ultrastructure

## Abstract

The first electron microscope was constructed in 1931. Several decades later, techniques were developed to allow the first ultrastructural analysis of microglia by transmission electron microscopy (EM). In the 50 years that followed, important roles of microglia have been identified, specifically due to the ultrastructural resolution currently available only with EM. In particular, the addition of electron-dense staining using immunohistochemical EM methods has allowed the identification of microglial cell bodies, as well as processes, which are difficult to recognize in EM, and to uncover their complex interactions with neurons and synapses. The ability to recognize neuronal, astrocytic, and oligodendrocytic compartments in the neuropil without any staining is another invaluable advantage of EM over light microscopy for studying intimate cell–cell contacts. The technique has been essential in defining microglial interactions with neurons and synapses, thus providing, among other discoveries, important insights into their roles in synaptic stripping and pruning *via* phagocytosis of extraneous synapses. Recent technological advances in EM including serial block-face imaging and focused-ion beam scanning EM have also facilitated automated acquisition of large tissue volumes required to reconstruct neuronal circuits in 3D at nanometer-resolution. These cutting-edge techniques which are now becoming increasingly available will further revolutionize the study of microglia across stages of the lifespan, brain regions, and contexts of health and disease. In this mini-review, we will focus on defining the distinctive ultrastructural features of microglia and the unique insights into their function that were provided by EM.

## Introduction

Microglia are the only immune cells that permanently reside in the brain. Originally believed to mediate inflammatory responses to infection (1), trauma (2), ischemia (3), or neurodegenerative disease (4), recent studies identified microglia as crucial actors in the proper development and maintenance of neuronal circuits (5). del Río-Hortega provided the original morphological description of microglia at the turn of the twentieth century, having modified Golgi’s silver stain to identify microglia (6). His manuscripts have recently been translated into English and annotated (7). Early research into microglial physiology prompted researchers to posit hypotheses that still hold true: microglia are phagocytic; they are capable of generating inflammation in response to infection; they may be responsible for some aspects of neurodegenerative disease; they originate outside of the brain and colonize it early in development (8). Between the early twentieth and twenty-first centuries microglia remained mainly uninvestigated as a cell type [reviewed in Ref. (9)], until Davalos et al. and Nimmerjahn et al. uncovered their incredibly dynamic processes in the adult brain under physiological conditions using two-photon (2p) microscopy (10, 11). Following this discovery and with the development of genetic tools to specifically identify microglia and their progeny (12–14), high throughput gene-expression analysis (15–18), and investigation into expression of cell surface receptors (19, 20), researchers have completed a whirlwind of studies in an attempt to unravel microglial roles in a myriad of healthy and disease processes (21). Recent developments in super-resolution and 2p microscopy have provided insight into microglial interaction with dendritic spines (22–24). However, genetic manipulations required for marker expression in neurons and microglia can induce cellular stress and impair normal functions (25–27). Electron microscopy (EM) can be used to investigate the unique ultrastructure of microglia and their relationship with synapses, and identify their phagocytic cargo without any immunohistochemical or genetic labeling. While super-resolution microscopy has surpassed the diffraction limit of light microscopy, its resolution is still insufficient to discern samples smaller than 50 nm, especially in the *z*-dimension, and requires specific labeling probes to prevent steric hindrance from influencing the resulting image (28). In this review, we will focus on the use of EM to unravel structural and functional mysteries of microglia and their interaction with healthy and diseased brain tissue.

## History and Development of EM

Electron microscopy utilizes focused electron beams to illuminate the subject of interest. Since an electron’s wavelength is up to 100,000 times shorter than a photon’s, EM is capable of resolving atomic structures, while most light microscopes are diffraction-limited to 500 nm resolution.

Hans Busch, a pioneer in the field of electron optics, laid the theoretical groundwork for EM by determining the motion of electrons in a magnetic field and the potential to focus electron beams (29). The first EM was invented by Knoll and Ruska in 1932, based on the Bush’s published theories (30). The first transmission electron microscope (TEM) functioned by projecting electrons through a thin sample and onto film, and investigating the regions of the sample that were electron-permissive versus electron-dense. Shortly after TEMs were developed, the first scanning electron microscope (SEM) was invented in 1940 (31). SEM differs from TEM as it visualizes electrons that are scattered off the surface of the specimen instead of electrons that pass through the specimen.

**Figure 1:**
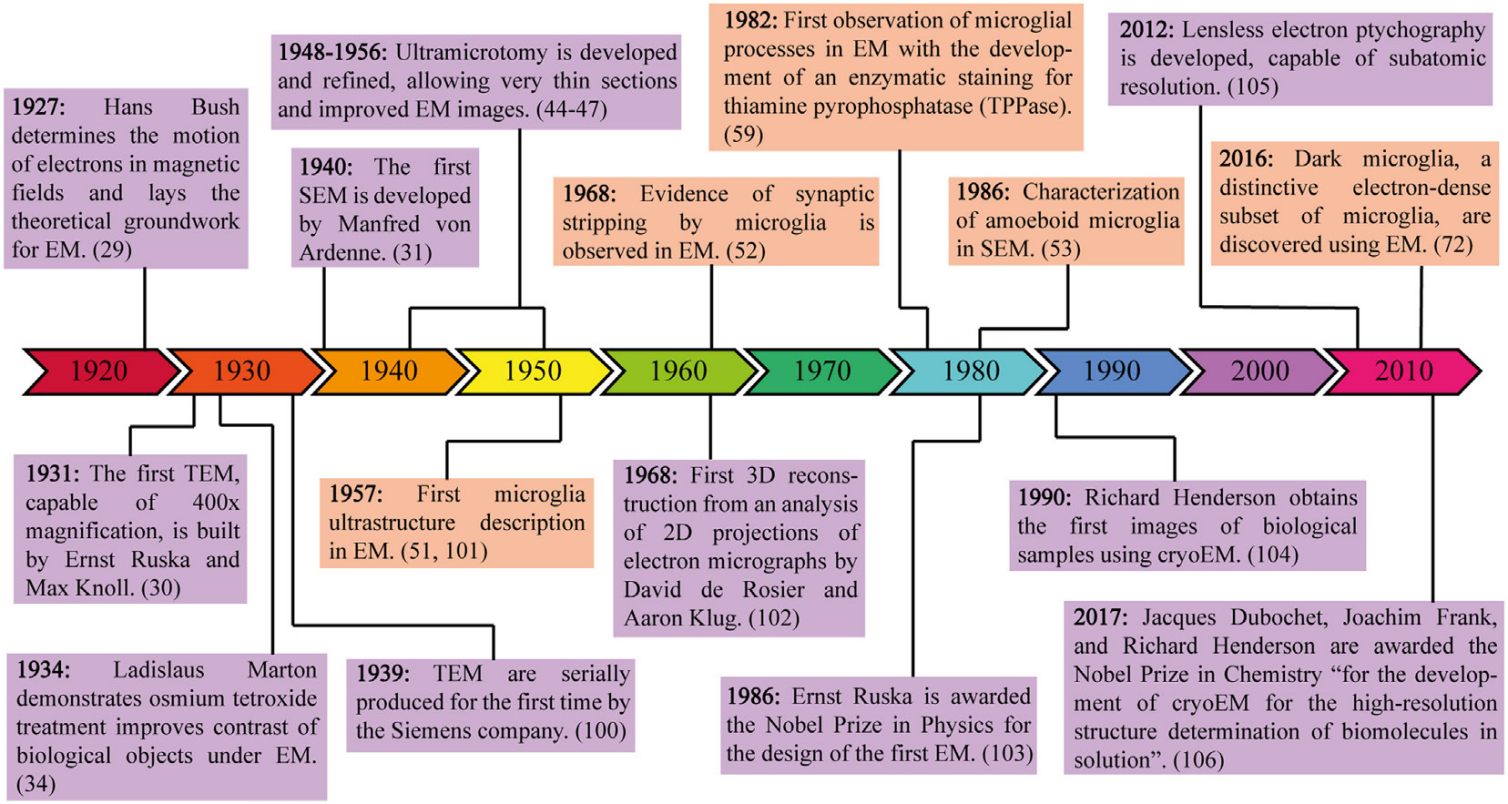
Milestones in electron microscopy (EM) engineering and discovery. This timeline highlights the major theoretical and experimental advances in EM, from the invention of the first electron microscope to the 2017 Nobel Prize in Chemistry for the discoveries leading to cryoEM. Purple frames contain information about the development of technology required for EM, while orange frames contain information about microglial discoveries made possible through the use of EM.

Over the following three decades, scientists perfected multiple ways to process and preserve biological samples in order to garner useful images of *in situ* tissue preparations (Figure 1). Aldehyde fixation cross-links proteins in tissues (32, 33), while osmium tetroxide fixation mainly preserves lipids and renders membranes electron-dense (34). The development of transcardiac perfusions provided fast delivery of fixatives to deep regions of the brain and other biological tissues, arresting any possible degradation that may have occurred in diffusion-dependent fixation techniques (35–37). However, using aldehydes or other fixatives results in tissue shrinkage and loss of extracellular space. This can be avoided by freeze substitution, a type of cryoEM: flash-freezing the tissue of interest followed by fixation performed at very low temperatures (38). Fixing the specimen in buffers that match the osmolarity of the tissue of interest can preserve extracellular space (39). Alternatively, if the specimen and chamber of the EM are kept below −140°C, samples can be visualized without any fixation (40, 41). Cell viability assays and staining for cell surface markers can be performed on live cells in suspension prior to deposition onto TEM grids and flash-freezing (42, 43).

Particularly important for TEM imaging was the development of ultramicrotomy, which allowed ultrathin (50–80 nm) sections to be cut from larger specimens, thus improving resolution and focus (44–47). These ultrathin sections allowed researchers to visualize ultrastructural images of various biological samples by capturing the transmitted electrons after they passed through the specimen onto films. The conventional protocol to prepare biological tissue for TEM is well explained by several groups (48–50).

## EM and Microglia

In 1957, the first ultrastructural image of microglia in the rat parietal cortex was published (51), and in 1968, TEM images showed microglia physically separating presynaptic terminals from postsynaptic dendrites or neuronal cell bodies, a term defined as synaptic stripping (52). The first TEM images of microglia uncovered clues to the dynamic nature of these cells, decades before 2p microscopy discovered their movements to survey the brain parenchyma in real-time. Cultured microglia investigated using SEM identified many tiny processes projecting directly from cell somas, and draw stark attention to the two-dimensional stressors placed on cells in culture (53). Pioneering studies in EM identified many unique characteristics of microglial cell bodies, before any cell-specific immunological studies were developed.

**Figure 2:**
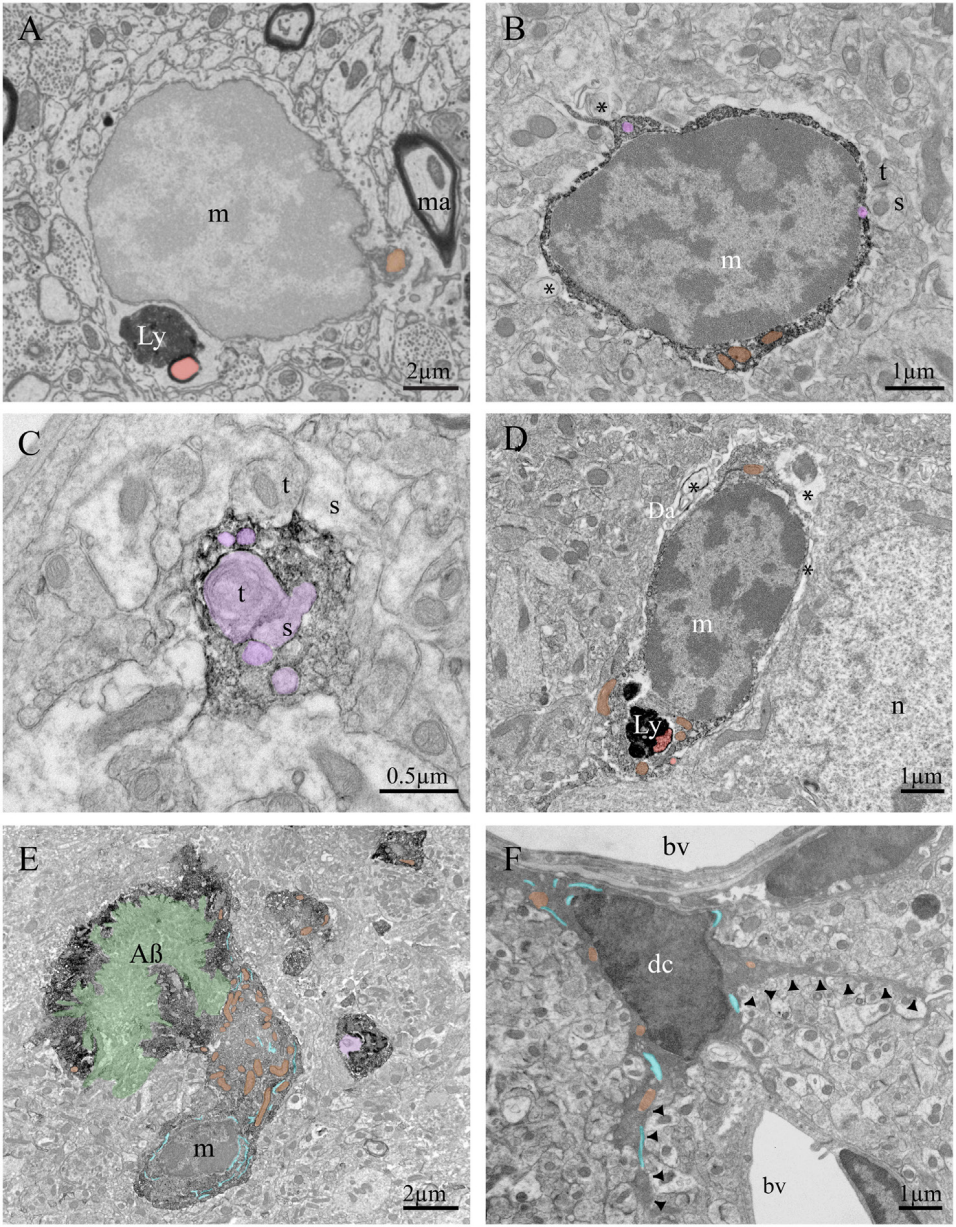
Ultrastructural features of murine brain microglia in health and disease. Example of microglia imaged using a focused-ion beam coupled with scanning electron microscope without any immunostaining **(A)**, containing lipofuscin granules (Ly) and a lipid body (Lb). Diaminobenzidine staining against ionized calcium-binding adapter molecule 1 (Iba1) creates a dark immunoprecipitate in the cytoplasm as shown by transmission electron microscopy (TEM) **(B–E)**. Iba1 staining allows identification of microglial processes in fractalkine receptor-knockout mice, for instance, allowing researchers to investigate their contacts with synaptic terminals and study phagocytic inclusions. **(B)** A microglial cell body in an APP-PS1 mouse is contacting a synapse between two axon terminals and a dendritic spine, as well as juxtaposing cellular debris. **(C)** A microglial process in a C57Bl/6 mouse contains several inclusions, notably an axon terminal making a synaptic contact on a dendritic spine. **(D)** A microglial cell body in a mouse model of Werner syndrome juxtaposes myelin debris and contains lipofuscin granules. **(E)** A microglial cell body in an APP-PS1 mouse is found in intimate contact with an amyloid beta plaque. **(F)** Example of dark microglia observed by TEM in a stressed fractalkine receptor-deficient mouse, characterized by its dark cytoplasm and thin processes projecting from the cell body (black arrowheads). Symbols and abbreviations: m, microglia; n, neuron; dc, dark microglia; t, axon terminal; s, dendritic spine; bv, blood vessel; Ly, lipofuscin; Da, degenerated axon; ma, myelinated axon; AB, amyloid-beta plaque. Asterisk (*) denotes evidence of cellular debris undergoing digestion in the extracellular space. Pseudocolor code: phagocytic inclusions = purple, examples of dilated endoplasmic reticulum = blue, examples of mitochondria = orange, amyloid-beta plaque = green, lipid bodies = red.

Microglial cell bodies can be discerned from those of other cell types by their small size (3–6 μm), electron-dense cytoplasm, and characteristically bean-shaped nuclei. They also display a distinct heterochromatin pattern. A thick, dark band of electron-dense heterochromatin is located near the nuclear envelope, with pockets of compact heterochromatin nets throughout the nucleus. These nets are often visualized as small islands of dark heterochromatin within a sea of more loosely packed, lighter euchromatin within the central part of the nucleus (54, 55). Microglial cell bodies have a very thin ring of cytoplasm separating their nuclei from their cell membranes, and contain few organelles within a single ultrathin section, but those visible are mostly mitochondria, long stretches of endoplasmic reticulum, Golgi saccules, and lysosomes (54, 56, 57). They are often phagocytic and contain lipidic inclusions, especially in older animals (58) (Figures 2A,D).

The development of microglial-specific stains compatible with EM has been a major aid in determining their functions *in situ*. Labeling microglial membranes and cytoplasm, originally with enzymatic reactions and more recently with immunoEM, allowed researchers to investigate microglial processes in animal models and human postmortem tissue (57, 59, 60). Current immunoEM studies utilize either diaminobenzidine or gold-conjugated antibodies (or colabeling using both) to deposit electron-dense precipitate and identify proteins of interest (22, 61). Ionized calcium-binding adapter molecule 1 (Iba1) is often used to identify microglia/macrophages within the brain (62). After much study using immunoEM to identify their main characteristics (22), trained researchers can identify microglial processes based solely on their unique ultrastructure. Microglia’s ramified projections are long, thin, and almost never contiguous with their cell bodies in ultrathin sections examined by TEM (Figures 2B,C). They are often in close, direct contact with neuronal cell bodies, or separated only by a very thin astrocytic process (22, 57, 59, 63). A single microglial process can contact multiple synaptic elements, and interacts with axon terminals, dendritic spines, perisynaptic astrocytic processes, and encircles parts of synaptic clefts (22, 63). Their processes often perform extracellular degradation, visible as pockets of extracellular space sometimes containing pinpoints of membrane degradation. They frequently contain vacuoles or multivesicular bodies, long stretches of endoplasmic reticulum, and phagocytic inclusions (Figure 2C).

Microglia promote proper neuronal wiring and activity, and EM studies were vital for discovering their role in development and maintenance of functional neuronal connections (21). Elegant EM studies demonstrated that glia (performing the functions of microglia) in *Drosophila* (64), macrophages and microglia in zebrafish (65), as well as microglia in rodents (66–68) phagocytose degenerating axonal tracts, axon terminal fragments, and dendritic spines during development of the thalamus, cerebral cortex, and hippocampus. Interestingly, no phagocytic interactions between microglia and synapses were identified in TEM studies of a mouse model of prion disease (69), although immunoEM was not performed and microglial processes may have been overlooked. Microglia also phagocytose putative neuronal debris following saponin-induced cholinergic cell death in rats (70). Sequential EM images are required to verify phagocytic cargo is fully enclosed within a microglial process and has been demonstrated for phagocytosis of synaptic elements by both microglia and astrocytes (22, 67, 71). Automation of sequential EM using knives or focused-ion beams inside SEM chambers can provide nanometer-scale resolution images of microglia in 3D.

Recent TEM studies have uncovered a new microglial phenotype, named dark microglia. These dark microglia share many ultrastructural characteristics (including cell size, immunohistochemical markers, and phagocytic phenotype) with healthy microglia, yet appear strikingly different under TEM. Their cell bodies can be quickly identified by their condensed, electron-dense cytoplasm that makes them appear as dark as mitochondria. Dark microglia display many signs of cellular stress, including nuclear and chromatin condensation and dilation of their endoplasmic reticulum (Figure 2F). Additionally, they are present in greater numbers in pathological contexts often associated with neuronal dystrophy and distress. They have been identified in the APP-PS1 mouse model of Alzheimer’s disease (AD), aged mice, animals subjected to social defeat stress, fractalkine receptor-deficient mice, and mouse models of schizophrenia (72, 73). They show reduced expression levels of some microglial markers, including Iba1, but are strongly immunopositive for others, including complement receptor subunit CD11b and microglia-specific antibody 4D4. Most dark microglia located near amyloid plaques in APP-PS1 mouse model express TREM2, though dark microglia in other disease models do not. While normal microglia rarely have contiguous processes attached to their somas in ultrathin sections, dark microglia show many long, thin processes encircling dystrophic neurons, wrapping around synaptic structures, investing themselves deep into amyloid beta plaques, and interacting with synapses in regions of high synaptic turnover (72). Although they display many signs of cellular stress, they have never been found expressing apoptotic or necrotic cell markers. Dark microglia are often located near blood vessels, which could imply a possible peripheral origin or perhaps a route of egress for the stressed cell to leave the brain parenchyma (Figure 2F). As there is not yet a definitive marker of dark microglia, they can only be investigated with EM, highlighting its relevance in modern microglial biology studies.

## Correlative Light and Electron Microscopy (CLEM)

The combination of both light microscopy and EM can be used to uncover more information than either technique individually. CLEM was first used in 1969. Silver staining originally described by Río-Hortega was used to identify and investigate microglia in light microscopy. After confirming microglial-specific staining, researchers investigated ultrathin sections under TEM and published the first description of microglial ultrastructure (54).

Electron microscopy is currently used to unravel details and variations in ultrastructure that cannot be investigated with light microscopy (Table 1). Light microscopy is often used to detect changes in microglial density and morphology in health and disease (74), and to identify particular regions of interest. After identifying a region of interest, such as one affected by hypoxia in stroke or amyloid-beta positive plaque-containing tissue in AD, EM can delve further into specific changes in microglial ultrastructure, cellular viability and stress, all without requiring further immunostaining markers (75, 76). EM can also reveal structures which are not otherwise visible, and discern subcellular localization of proteins and mRNA using immunostaining, *in situ* hybridization, or *in situ* RT-PCR (57, 77, 78). EM was recently used to clarify microglial process fragmentation observed with light microscopy in postmortem human tissue from an individual suffering from AD. Unexpectedly, EM studies revealed no fragmentation as the two parts of the microglial process were linked by a cytoplasmic bridge, thus invalidating the original hypothesis (79).

**Table 1:** Types of EM.

Type of EM	Typical sample preparation	Maximal resolution	Advantages	Disadvantages
Transmission electron microscopy (TEM) (49)	– Fixation with aldehydes and plastic resin embedding – Manually cut ultramicrotomy (thin sections of 50–80 nm stored on metal grids)	Nanometer resolution in *x, y* Resolution in *z* limited by section thickness	– Tissue can be archived and reimaged – Block of tissue may be saved and recut – Highest resolution and magnification – Osmium fixation is not required	– Biological specimens must be fixed with gluteraldehyde or acrolein – Low throughput – Electron beam can cause deformation of ultrathin tissue sections – Smaller magnification range (680× to greater than 30,000×)
Scanning transmission electron microscopy (STEM) (49)	– Fixation with aldehydes, strong post-fixation with osmium (OTO), and plastic resin embedding – Manually cut ultramicrotomy (thin sections of 50–80 nm stored on metal grids)	Nanometer resolution in *x, y* Resolution in *z* limited by section thickness	– Tissue can be archived and reimaged – Block of tissue may be saved and recut – Faster imaging throughput than traditional TEM – Large magnification range (20× to greater than 30,000×)	– Biological specimens must be fixed with gluteraldehyde or acrolein – Stronger osmium fixation required than traditional TEM – Electron beam can cause deformation of ultrathin tissue sections – Risk of tissue destruction is higher than with traditional TEM
Scanning electron microscopy (SEM) (80)	– Dehydration – Strong post-fixation with osmium (OTO) if material contrast imaging is desired – Entire specimen (entire insect, dissected organ, etc.) mounted on a stub of metal with adhesive – Coated with a conductive metal	Nanometer resolution in *x, y*, and *z* for surface topography	– Tissue can be archived and reimaged – Large magnification range (20× to greater than 30,000×) – Can create images of up to several cm^3^, which provides a good representation of the 3D shape of the specimen – Secondary electron detector measures surface topography – Backscatter electron detector measures material contrast (i.e., cell membrane versus cytoplasm)	– Biological specimens must be fixed with gluteraldehyde or acrolein – Image is created using scattered electrons and limited to the surface of the specimen
Scanning electron microscopy with array tomography (81)	– Fixation with aldehydes, strong post-fixation with osmium (OTO), and plastic resin embedding – Manually or automatically cut serial sections ultramicrotomy (thin sections of 50–80 nm stored on silicon chips or magnetic tape)	Nanometer resolution in *x, y* Resolution in *z* limited by section thickness	– Tissue can be archived and reimaged – Image large and serial sections – Large magnification range (20× to greater than 30,000×) – Compatible with correlative light-EM imaging – No deformation of tissue, making serial reconstruction simpler	– Serial section cutting and collecting is technically challenging – Stronger fixation required for proper material contrast
Focused-ion beam–scanning electron microscopy (FIB–SEM) (82)	– Fixation with aldehydes, strong post-fixation with osmium (OTO), and plastic resin embedding – Prepared tissue specimen (3–10 mm^2^ wide × 3–10 mm^2^ tall × 50–75 μm thick) mounted on a stub of metal with adhesive – Coated with a conductive metal	Nanometer resolution in *x, y* Up to 5 nm resolution in *z* (83)	– Nanometer resolution (less than 5 nm per pixel) in all three dimensions – Simplest serial image reconstruction	– The entire tissue block must be mounted and cannot be resectioned – Limited to a very small area, usually less than 15 μm × 15 μm – Smaller magnification range (400× to greater than 30,000×) – The sample is destroyed as it is imaged and cannot be reimaged
CryoTEM (84) CryoSEM (84)	– High-pressure freezing – Manually or automatically cut sections using cryo-ultramicrotomy (40–100 nm thick)	Nanometer resolution in *x, y* Resolution in *z* limited to section thickness	– No fixation required – Allows imaging of specimens in a native-like state	– Technically challenging – The sample must be flash-frozen to preserve native protein folding – The sample must remain frozen through entire process

*Table of the major types of electron microscopy (EM) described in this mini-review, highlighting sample preparation, maximal resolution, magnification power, and advantages and disadvantages to each technique. Typical sample preparation is provided for each method, but fixation with aldehydes can be avoided if the researchers instead flash-freeze samples and perform freeze-substitution following sample collection*.

Light microscopy can also be performed on living cells prior to investigation with EM to tie temporal information to ultrastructural resolution. The technique used is a specific type of CLEM. Live imaging using 2p microscopy studies cellular relationships, interactions with the surrounding environment, and intracellular dynamics in real-time; but lacks complex ultrastructural information. These imaging techniques are also limited to genetically encoded or virally introduced cell-specific fluorescent markers, which may introduce phenotypic changes on their own (26). EM can uncover structural information, but the specimen must be fixed (or flash-frozen for cryoEM), and can only be investigated as a snapshot moment in time. CLEM integrates imaging of fluorescent proteins in live cells with the ultrastructural resolution of EM. After live-cell imaging is performed, various fixation and staining techniques can be employed to investigate ultrastructure in the same tissues. van Rijnsoever and colleagues used CLEM to study the endolysosomal system by confocal microscopy followed by cryoEM to image protein structures with nanometer resolution (85). This technique could be used to obtain insight into microglial proteins, phagocytic machinery, and organelle biogenesis.

It is also possible to combine EM with 2p microscopy. While 2p studies allow investigation of live microglial interactions with nearby neurons, EM performed afterward can study the intimate contacts between microglia and synaptic elements, and their changes in response to various behavioral experiences and pathologies (22, 63, 86–88). Light microscopy also informs EM studies, making it much easier to solve needle-in-haystack problems and identify rare events within the neuropil. For example, an Alzheimer’s study injected animals with methoxy-X04, a blood–brain barrier permeable amyloid-beta fluorescent marker. Researchers then selected sections containing the region of interest known to contain amyloid-beta, thus increasing the likelihood of finding plaque-associated microglia in ultrathin sections (Figure 2E) (50). Similarly, fluorescent microscopy can be used to target a specific microglial population to be analyzed in EM. Bechmann and Nitsch fluorescently labeled axons prior to performing entorhinal lesions and traced the clearance of degenerating tissue by identifying the fluorescent compound within nearby microglia. By focusing EM studies on regions containing fluorescently labeled microglia, they were able to investigate the subpopulation of microglial cells which had phagocytosed degenerating axons (89).

Another CLEM technique is the use of light microscopy in correlation with cryoEM. A study utilizing both techniques recently discovered the native folding of herpes simplex virus as it moved throughout axons. 3D visualization permits analysis of vesicle fusion and actin bond formation (90). CryoEM was also recently used to image Golgi apparati in two different conformations within neurons (91) and investigate minor changes in ultrastructure following intracerebral injections (a common technique used to introduce vectors into mouse models) (92). Cryo-fixation preserves extracellular space, especially notable at synapses and blood vessels (93). This method could be used to determine native folding and unfolding of proteins within microglia, to better understand their morphological and functional changes in various disease conditions.

## The Future of EM and Microglia

The past 15 years have seen a whirlwind in EM development. Previously, when investigating 3D ultrastructure, serial ultrathin sections were manually cut and collected at the ultramicrotome, imaged individually onto film under a TEM, and painstakingly reoriented and collated prior to analysis (94). As digital imaging improved, TEMs were outfitted with digital cameras allowing for faster imaging, but the electron beam of the TEM could still deform ultrathin sections, making perfect alignment of sequential sections almost impossible. Developments in SEM opened the door for array tomography studies on ribbons of serial ultrathin sections, allowing CLEM on the same tissue, and solving the problem of deformation introduced when using TEM (81).

The first major revolution in 3D ultrastructure imaging came when Denk and Horstmann engineered a working diamond knife into the chamber of a SEM to perform serial block-face scanning electron microscope (SBEM) (95). With this approach, the block face is imaged, a 50-nm section is cut away, followed by another image. Peddie and Collinson recently reviewed the many types of 3D EM and its applications to biological tissues (96). A decade after SBEM was invented, it allowed researchers to confirm microglial synaptic stripping (97). Research in a mouse model of multiple sclerosis used SBEM to unravel different roles of microglia *versus* infiltrating monocytes very early in the disease. The authors performed SBEM and differentiated resident microglia from invading myeloid cells by their ultrastructural differences (changes in mitochondrial makeup, nuclear shape, and presence of osmiophilic granules) in order to determine that demyelination in experimental autoimmune encephalitis is initially performed by invading monocytes, while resident microglia did not contribute to the early stages of inflammation (98). More recently, focused-ion beam coupled with SEM (FIB-SEM) has improved resolution from 50 nm to less than 10 nm in the *z*-dimension (99). By employing a focused-ion beam to atomize a very thin layer from a small (usually less than 500 μm^2^) area, researchers may image at 5 nm resolution in *x, y*, and *z* (82).

Both SBEM and FIB-SEM are capable of investigating neuronal ultrastructure, and can follow a single process through several microns of neuropil, but FIB-SEM is also capable of resolving synaptic vesicles, lysosomes, and phagosomes in three dimensions. If the FIB-SEM process began within a microglial cell body, researchers could trace fine microglial processes through several microns of neuropil, without having to perform immunoEM. This offers a better chance to investigate lipidic inclusions and other pathological changes in organelles obscured by electron-dense precipitates used in immunoEM.

In addition to technological advances in both SEM and TEM, the rapid development of cryoEM techniques described here could uncover native protein structures within microglia. It could additionally pave the way for discoveries into the snapshot of microglial–neuron and microglia–glia interactions without requiring fixatives, and without the corresponding tissue deformation that occurs with rapid fixation currently required to preserve ultrastructure. While fixatives and ultrathin sections required for EM are not compatible with post-imaging analysis of RNA or proteins, future iterations of CLEM (perhaps cryoCLEM) and advances in single-cell mRNA isolation may be able to isolate subcellular tissue fractions for further analysis. Armed with these new tools, biologists may investigate the complex interactions between glia and neurons in a number of diseases. The unique nature of EM allows researchers to characterize unique ultrastructural characteristics of microglia and other immune cells, and uncover possible paths for therapeutic intervention.

## Author Contributions

JS and MET conceived the ideas and drafted the manuscript to which all authors contributed. KP and FG-I created the figures. All authors read and approved the final version of the manuscript.

## Conflict of Interest Statement

The authors declare that the research was conducted in the absence of any commercial or financial relationships that could be construed as a potential conflict of interest. The handling Editor declared a past co-authorship with one of the authors MET.

## Abbreviations

2p, two-photon; EM, electron microscopy; TEM, transmission electron microscope; SEM, scanning electron microscope; DAB, diaminobenzidine; Iba1, ionized calcium-binding adapter molecule 1; CLEM, correlative light and electron microscopy; AD, Alzheimer’s disease; SBEM, serial block-face scanning electron microscope; FIB-SEM, focused-ion beam coupled with scanning electron microscope.
